# Comparison of Enzyme Secretion and Ferulic Acid Production by *Escherichia coli* Expressing Different *Lactobacillus* Feruloyl Esterases

**DOI:** 10.3389/fmicb.2020.568716

**Published:** 2020-11-03

**Authors:** Zhenshang Xu, Jian Kong, Susu Zhang, Ting Wang, Xinli Liu

**Affiliations:** ^1^State Key Laboratory of Biobased Material and Green Papermaking, Qilu University of Technology, Shandong Academy of Science, Jinan, China; ^2^Shandong Provincial Key Laboratory of Microbial Engineering, Department of Bioengineering, Qilu University of Technology, Shandong Academy of Science, Jinan, China; ^3^State Key Laboratory of Microbial Technology, Shandong University, Qingdao, China

**Keywords:** *Lactobacillus*, feruloyl esterase, secretion, *Escherichia coli*, ferulic acid

## Abstract

Construction of recombinant *Escherichia coli* strains carrying feruloyl esterase genes for secretory expression offers an attractive way to facilitate enzyme purification and one-step production of ferulic acid from agricultural waste. A total of 10 feruloyl esterases derived from nine *Lactobacillus* species were expressed in *E. coli* BL21 (DE3) to investigate their secretion and ferulic acid production. Extracellular activity determination showed all these *Lactobacillus* feruloyl esterases could be secreted out of *E. coli* cells. However, protein analysis indicated that they could be classified as three types. The first type presented a low secretion level, including feruloyl esterases derived from *Lactobacillus acidophilus* and *Lactobacillus johnsonii*. The second type showed a high secretion level, including feruloyl esterases derived from *Lactobacillus amylovorus*, *Lactobacillus crispatus*, *Lactobacillus gasseri*, and *Lactobacillus helveticus*. The third type also behaved a high secretion level but easy degradation, including feruloyl esterases derived from *Lactobacillus farciminis*, *Lactobacillus fermentum*, and *Lactobacillus reuteri*. Moreover, these recombinant *E. coli* strains could directly release ferulic acid from agricultural waste. The highest yield was 140 μg on the basis of 0.1 g de-starched wheat bran by using *E. coli* expressed *L. amylovorus* feruloyl esterase. These results provided a solid basis for the production of feruloyl esterase and ferulic acid.

## Introduction

Feruloyl esterase (E.C. 3.1.1.73), belonging to the hemicellulase family, is a type of hydrolase capable of degrading the ester bond between the ferulic acid and lignin in the cell wall of plants. It acts in conjunction with other cellulases and hemicellulases to synergistically open the crosslinked network structures of cell walls (Fazary et al., 2009; Várnai et al., 2014). This feature endows its applications in many areas, such as feed additives and pulp and paper industries. Due to the structural complexity of the materials in the feed, they cannot be fully degraded and utilized after being ingested by animals, leading to the low utilization rate. The feruloyl esterase is able to break the crosslinking between cellulose, hemicellulose, and lignin, thus making the feed becoming sparse. The feruloyl esterase-treated feed is easy to be digested and absorbed by livestock (Lynch et al., 2015). In the pulp and paper making industry, the usage of feruloyl esterase avoids the environmental pollution and energy consumption which is caused by the traditional chemical methods. The composition of the paper is mainly cellulose and hemicellulose. Therefore, the lignin component in the plant cell walls needs to be removed. This process is mainly attributed to feruloyl esterase (Record et al., 2003). Furthermore, feruloyl esterase has the reaction ability of esterification or transesterification, indicating that the ester bond between phenolic acid and sugar can be synthetized by a biological enzymatic method. The produced esters have the promising potential for application as antibacterial, antiviral, and anti-inflammatory drugs (Chong et al., 2019).

Nowadays, feruloyl esterases have been found in a variety of microorganisms including bacteria and fungi (Udatha et al., 2011; Wang et al., 2016). They showed different coding sequences, protein structures, physicochemical properties, and catalytic activities. For characterization of a feruloyl esterase, the routine experimental procedures contain cloning of the coding gene, heterologous expression of the enzyme in recombinant strain, and then purification of the feruloyl esterase. *Escherichia coli* expression systems are commonly used for prokaryotic feruloyl esterase expression (Xu et al., 2017). This system is the most well-researched, classical, high-efficiency heterologous expression system for prokaryotic genes. *Escherichia coli* has been used as a cell factory to produce a considerable number of enzymes and medical proteins due to its clear genetic background, simple and easy operation, and high protein yield (Khan et al., 2013). However, because of its internal and external bilayer membrane structure, the secretory expression of protein becomes a problem in *E. coli*. The feruloyl esterase produced by engineering *E. coli* is usually located in the cytoplasm. A complicated purification process is needed to obtain the desired product for further study (Rakotoarivonina et al., 2011). Nevertheless, there had been very few reports concerning the fact that the recombinant proteins were detected extracellularly when heterologous proteins were expressed in *E. coli*, such as β-xylosidase, cellulase, and cutinase (Teng et al., 2011; Gao et al., 2015; Su et al., 2015). In our previous study, we fortunately found that the *Lactobacillus crispatus* feruloyl esterase also could be secreted into the extracellular environment of *E. coli* (Xu et al., 2019). Considering that the feruloyl esterase coding genes widely exist in different *Lactobacillus* species, whether these feruloyl esterases share the same secretory characteristic is worth investigation.

Another benefit of the extracellular secretory feruloyl esterase is its direct use for ferulic acid production. Ferulic acid, also known as 4-hydroxy 3-methoxycinnamic acid, is crosslinked with other components of the cell wall of plants. Feruloyl esterase hydrolyzes ester bonds to release ferulic acid (Deng et al., 2019). Studies have shown that ferulic acid has many important biological effects. As an antioxidant, it can remove various free radicals, thus functioning as an anti-aging regulating agent. Furthermore, ferulic acid has obvious effects in reducing inflammation, promoting wound healing and anti-tumor (Eitsuka et al., 2016; Zheng et al., 2019). Therefore, a variety of functional foods can be developed by using ferulic acid. Moreover, ferulic acid is the substrate for specific microorganisms to produce vanillin, which is used as a spice in the food and cosmetics industries (Banerjee and Chattopadhyay, 2019). At present, feruloyl esterase alone or in combination with other enzymes such as xylanase is applied to extract ferulic acid from the crop by-products including wheat bran and rice bran. The used feruloyl esterase is usually obtained from the procedures of expression in a heterologous host and subsequent purification (Phuengmaung et al., 2019). Several studies were conducted, using the naturally extracellular feruloyl esterase of microorganisms to produce ferulic acid, but the expression level of the enzyme is generally low (Pérez-Rodríguez et al., 2016; Long et al., 2018). The utilization of high-efficiency expression systems to mass produce feruloyl esterase and secrete it extracellularly is a very attractive method for the production of ferulic acid.

In the present study, a total of 10 feruloyl esterases derived from nine *Lactobacillus* species were used to analyze their commonness and compare their differences when heterologously expressed in *E. coli*. These feruloyl esterase coding genes were cloned and expressed in *E. coli* BL21 (DE3), respectively. The changes of extracellular feruloyl esterase activities were measured, and the profiles of cytoplasmic and extracellular protein bands were visualized. Moreover, these recombinant *E. coli* strains were directly used for ferulic acid production in a medium containing de-starched wheat bran.

## Materials and Methods

### Strains, Plasmids, Culture Conditions, and Chemicals

The *Lactobacillus* strains of *L. acidophilus*, *L. amylovorus*, *L. crispatus*, *L. farciminis*, *L. fermentum*, *L. gasseri*, *L. helveticus*, *L. johnsonii*, and *L. reuteri* were anaerobically cultured at 37°C in MRS (De Man, Rogosa and Sharpe) medium, which is composed (per liter) of tryptone, 10 g; yeast extract, 5 g; glucose, 20 g; ammonium citrate, 0.58 g; MnSO_4_, 0.25 g; CH_3_COONa·3H_2_O, 3.12 g; Na_2_HPO_4_, 1.63 g; CH_3_COOK, 2.25 g; beef extract, 10 g; and Tween-80, 1 ml. The strain *E. coli* DH5α was used for recombinant plasmids construction. The strain *E. coli* BL21 (DE3) was used for heterologous feruloyl esterases expression. These two strains were aerobically cultivated in LB (Luria-Bertani) broth containing 10 g/L typtone, 5 g/L yeast extract, and 10 g/L NaCl. Plasmid pET-22b (Novagen, Madison, United States) was used for the ligation of feruloyl esterase genes. When the pET-22b and pET-22b-derivative plasmids were transformed into *E. coli*, the LB medium supplemented with ampicillin at a final concentration of 100 μg/ml was used. To produce ferulic acid from the agricultural waste, the recombinant *E. coli* BL21 (DE3) strains were cultured in LB broth supplemented with 2% (w/v) de-starched wheat bran.

Ferulic acid and ethyl ferulate were bought from Sigma Chemicals Industries., Ltd. (SanFrancisco, United States). Para-nitrophenyl ferulate (ρNPF) was procured from Shandong Chambroad Holding Co., Ltd. (Shandong, China). Bacterial genomic DNA extraction kit was purchased from Tiangen biotech Co., Ltd. (Beijing, China), and gel extraction kit, plasmid extraction kit, and cycle pure kit were purchased from Omega Bio-tek (Atlanta, United States). These kits were used by following the manufacture’s recommended protocols. Phanta max super-fidelity DNA polymerase and Exnase®II were purchased from Vazyme Biotech Co., Ltd. (Nanjing, China). Restriction enzymes were purchased from TaKaRa Biotech Co., Ltd. (Tokyo, Japan). The wheat bran was procured from a local mill of Jinan city (Shandong, China). All other regents were bought from Solarbio Science and Technology Co., Ltd. (Beijing, China).

### Cloning and Expression of *Lactobacillus* Feruloyl Esterases

The *Lactobacillus* strains were cultivated in MRS medium at 37°C for 12 h. The cells were collected by centrifugation at 6,000 × *g* for 5 min and washed twice by sterile water. Then the genomic DNA of these *Lactobacillus* strains were extracted by using bacterial genomic extraction kit as described above. The amount and quality of the obtained DNAs were determined with a microspectrophotometer (Eppendorf, Hamburg, Germany), and then stored at −20°C until to use. Based on the related reports and the genome sequences deposited in NCBI database, the primer sets were designed for amplification of these *Lactobacillus* feruloyl esterase coding genes, respectively. As shown in Supplementary Table S1, the nucleotides pairing with the feruloyl esterase gene sequence are in uppercase letter, and the nucleotides pairing with the pET-22b vector are in lowercase letters. The PCR amplification procedure contained an initial denaturation at 95°C for 3 min, followed by 30 cycles each of denaturation at 95°C for 30 s, annealing at 50°C for 30 s, extension at 72°C for 45 s, and then a final extension at 72°C for 5 min. The obtained feruloyl esterase genes were extracted from the gel after electrophoresis. The pET-22b vector was digested by NdeI and XhoI, and then purified. To ligate the gene into pET-22b, the vector and fragment were mixed with the molar ratio of 2:1, and the Exnase®II was used to activate the homologous recombination. After treatment at 37°C for 30 min, the reaction mixture was transformed into the *E. coli* DH5α competent cells using a heat shock method. The correct transformants were selected by colony PCR, and their plasmids were extracted and sequenced in Sangon Biotechnology Co. Ltd. (Shanghai, China). The putative signal peptides of these feruloyl esterases were predicted by using the signalP program^1^ and TatP program.^2^

The pET-22b vector and the generated pET-22b-derivative plasmids were further transformed into *E. coli* BL21 (DE3) cells by heat shock method. The transformants were picked up and inoculated into LB medium supplemented with 100 μg/ml ampicillin and cultivated at 37°C in a shaker at 200 rpm. To express the feruloyl esterases, the inducer isopropyl-β-D-thiogalactopyranoside (IPTG) at a final concentration of 0.5 mM was added into the cultures when the growth of *E. coli* cells reached an OD_600_ of 0.5. After induction, these *E. coli* strains were incubated at 25, 30, and 37°C for 12 h to explore the optimum induction temperature. To monitor the changes of enzymatic activity and protein production, the incubation was continued for 72 h at 37°C. The samples were taken at time intervals of 4, 8, 12, 24, 36, 48, 60, and 72 h. *Escherichia coli* BL21 (DE3) containing pET-22b was used as negative control.

### Feruloyl Esterase Activity Determination

The LB plate-based assay was conducted to detect the feruloyl esterase activity of the recombinant strains and extracellularly secretory component. At the plate-pouring stage, 100 μg/ml ampicillin, 0.5 mM IPTG, and 6.7 mM ethyl ferulate (dissolved in dimethylformamide) were added into LB medium and fully mixed. The recombinant *E. coli* BL21 (DE3) strains were inoculated in the plates and then incubated at 37°C for 18 h to detect the expression and activity of heterologous feruloyl esterases. To investigate whether these feruloyl esterases could be secreted out of the recombinant *E. coli* cells, an Oxford cup-based experiment was carried out. These *E. coli* strains were cultivated to an OD_600_ of 0.6 in a tube containing 5 ml LB broth, and then induced by 0.5 mM IPTG to produce feruloyl esterases for 12 h at 37°C. Then, the culture broth was centrifuged at 6,000 × *g*, 4°C for 5 min, and the supernatant was collected and filtered by 0.22-μm filter to obtain the cell-free culture supernatant. A volume of 200 μl cell-free culture supernatant was added into the Oxford cup placed in the plate to preliminarily detect the extracellular feruloyl esterase activity. All these plates were incubated at 37°C for 6 h, and the formed halo was observed and photographed.

The substrate ρNPF, which could be hydrolyzed by feruloyl esterase to produce the ρ-nitrophenol with a yellow color, was used to quantitatively determine the feruloyl esterase activity. The 1 mM substrate solution was prepared by adding 25 mM ρNPF (dissolved in dimethyl sulfoxide) into sodium phosphate buffer (100 mM, pH 7.0) which was supplemented with Tween-80 (1%, v/v) previously. Then, 100 μl of sample was mixed with 900 μl substrate solution to initialize the reaction. After incubation at 37°C for 10 min, 1 ml of acetic acid solution (50%, v/v) was added into the mixture to terminate the reaction. Meanwhile, the control experiments were performed using the inactivated sample. The released ρ-nitrophenol was determined at 410 nm using a spectrophotometer (Eppendorf, Hamburg, Germany). At the conditions described above, the required enzyme amount to produce 1 μmol ρ-nitrophenol in 1 min was calculated as one unit (U) of feruloyl esterase activity.

### Protein Analysis

The whole cell, cytoplasmic component, and extracellular component of *E. coli* BL21 (DE3) were prepared as follows. The recombinant strain cultures after induction were centrifugated at 6,000 × *g*, 4°C for 5 min to separate the cells and supernatant. The supernatant was filtered through a 0.22-μm filter, and represented the extracellular component. The harvested cells were resuspended in an equal volume of sodium phosphate buffer (100 mM, pH 7.0). This represented the whole cell of recombinant *E. coli*. An ultrasonic breaker (Tenlin, Jiangsu, China) was used to break the cell suspension with the conditions of power at 400 w, pulse 5 s, pause 5 s, and cycle 49 at 4°C. The cell-free extract was obtained by centrifugation at 17,400 × *g*, 4°C for 10 min and filtering through a 0.22-μm filter, representing the cytoplasmic component.

Sodium dodecyl sulfate-polyacrylamide gel electrophoresis (SDS-PAGE) was used to detect the protein bands of the whole cell, cytoplasmic component, and extracellular component of *E. coli*. All the components were mixed with 5 × SDS-PAGE loading buffer and then boiled for 10 min. The gel was composed of a 5% acrylamide stacking gel and a 12% acrylamide separating gel. After electrophoresis, the Coomassie brilliant blue staining solution was applied to visualize the protein bands. The unstained protein molecular weight marker SM0431 (Fermentas, Vilnius, Lithuania) was used as a standard to determine the molecular mass. The extracellular protein contents of *E. coli* cells cultured in LB medium were estimated by the Bradford protein assay in which the bovine serum albumin was used as a standard (Cheng et al., 2016).

For western blot analysis, the extracellular component was concentrated 10-fold by using trichloroacetic acid and applied for SDS-PAGE. The separated proteins were electro-transferred to a PVDF membrane (Millipore, Massachusetts, United States) at 250 mA for 90 min. After blocking with 5% non-fat milk for 3 h, the membrane was incubated with rabbit anti-His tag antibody (BIOSS, Beijing, China) at a dilution of 1:1000 for 90 min, and subsequently washed with TBST buffer (20 mM pH 7.4 Tris-HCl, 500 mM NaCl, and 0.01% Tween 20) and incubated with horseradish-peroxidase labeled goat anti-rabbit IgG (Servicebio, Wuhan, China) at a dilution of 1:3000 for 1 h. The bands were visualized by using ECL (Solarbio, Beijing, China) and photographed.

### Release of Ferulic Acid From De-Starched Wheat Bran

The ferulic acid releasing ability by these recombinant strains expressing *Lactobacillus* feruloyl esterases was investigated using de-starched wheat bran as substrate. A previously reported method was performed to prepare the de-starched wheat bran (Donaghy et al., 2000). In brief, 100 g fresh wheat bran was treated with amylase (0.3%, w/v) at 65°C for 30 min, and then with papain (0.3%, w/v) at 55°C for 45 min. The reaction mixture was boiled for 20 min to inactivate these enzymes. After centrifugation, the wheat bran was collected and washed repeatedly using distilled water to remove the starch completely. Subsequently, the de-starched wheat bran was dried to constant weight at 80°C and milled to passing a 60-mesh sieve. For ferulic acid production, the medium was prepared by adding 0.1 g de-starched wheat bran into a tube containing 5 ml LB broth, and then autoclaved. The recombinant *E. coli* strains were inoculated in the medium and cultivated at 37°C in a shaker at 200 rpm. The culture samples were taken out after 72 h induction by IPTG, and analyzed by high performance liquid chromatography (HPLC) as described below. To evaluate secretory feruloyl esterases on the released ferulic acid exactly, the recombinant strains were cultivated in tubes containing 5 ml LB broth. After induction cultures were centrifugated at 6,000 × *g*, 4°C for 5 min when the extracellular feruloyl esterase reached the maximum. The supernatant was filtered through a 0.22-μm filter, and added into a pre-autoclaved tube containing 0.1 g wheat de-starched wheat bran. The tubes were then shaken at 37°C until 72 h for HPLC analysis. The samples were boiled for 30 min and centrifuged at 10,000 × *g* for 15 min. The supernatant was harvested and filtered through a 0.22-μm filter before HPLC analysis.

The HPLC (Shimadzu, Kyoto, Japan) was equipped with a CBM-20A communications bus module, a LC-20AT pump, a SIL-20A auto sampler, a CTO-10A column oven, a reversed-phase WondaCract ODS-2 C18 cartridge, and a SPD-M10Avp photodiode array detector. This system was eluted by a mobile phase (methanol, water, and acetic acid as a ratio of 50:49.5:0.5) with a flow rate of 1 ml/min at 30°C. Absorbance of the eluent was monitored at 320 nm. The standard ferulic acid was used for qualitative and quantitative analysis of the samples.

### Statistical Analysis

Each value is expressed as mean ± SD (*n* = 3). All statistical procedures were performed using the statistical packages for the social sciences (SPSS).

## Results

### Expression of *Lactobacillus* Feruloyl Esterases in *E. coli*

The feruloyl esterase coding genes widely exist in the genome of a variety of lactic acid bacteria. In the present study, nine *Lactobacillus* strains belonging to *L. acidophilus*, *L. amylovorus*, *L. crispatus*, *L. farciminis*, *L. fermentum*, *L. gasseri*, *L. helveticus*, *L. johnsonii*, and *L. reuteri* were used to compare their feruloyl esterases. Especially, *L. johnsonii* could produce two feruloyl esterases. Primer sets were designed to amplify the feruloyl esterase coding genes. To reduce the redundant amino acid sequences derived from the expression vector, an *in vitro* homologous recombination method that relied on homologous sequences was used to ligate these genes into pET-22b vector. The recombinant plasmids for feruloyl esterases production were presented in Table 1. A plasmid pET22b-FaeLcr constructed previously was also used in the present study as a positive control and for comparative analysis (Xu et al., 2019). The 10 enzymes shared sequence similarity in the range of 44–87%, indicating that they had a certain degree of homology (data not shown). Furthermore, bioinformatic analysis by SignalP and TatP revealed that all these feruloyl esterases did not contain any predictable signal peptide sequences.

**Table 1:** Plasmids used in this study.

Plasmids	Characteristics	References
pET-22b	Amp^r^, expression vector for heterologous protein production in *E. coli*	Novagen
pET22b-FaeLac	Amp^r^, pET-22b vector ligated with feruloyl esterase gene of *L. acidophilus*	This study
pET22b-FaeLam	Amp^r^, pET-22b vector ligated with feruloyl esterase gene of *L. amylovorus*	This study
pET22b-FaeLcr	Amp^r^, pET-22b vector ligated with feruloyl esterase gene of *L. crispatus*	Xu et al., 2019
pET22b-FaeLfa	Amp^r^, pET-22b vector ligated with feruloyl esterase gene of *L. farciminis*	This study
pET22b-FaeLfe	Amp^r^, pET-22b vector ligated with feruloyl esterase gene of *L. fermentum*	This study
pET22b-FaeLga	Amp^r^, pET-22b vector ligated with feruloyl esterase gene of *L. gasseri*	This study
pET22b-FaeLhe	Amp^r^, pET-22b vector ligated with feruloyl esterase gene of *L. helveticus*	This study
pET22b-FaeLjo1	Amp^r^, pET-22b vector ligated with one feruloyl esterase gene of *L. johnsonii*	This study
pET22b-FaeLjo2	Amp^r^, pET-22b vector ligated with the other feruloyl esterase gene of *L. johnsonii*	This study
pET22b-FaeLre	Amp^r^, pET-22b vector ligated with feruloyl esterase gene of *L. reuteri*	This study

These pET-22b-deritative plasmids containing feruloyl esterase coding genes were further transformed into *E. coli* BL21 (DE3) for enzymes expression. The produced proteins were named as FaeLac (from *L. acidophilus*), FaeLam (from *L. amylovorus*), FaeLcr (from *L. crispatus*), FaeLfa (from *L. farciminis*), FaeLfe (from *L. fermentum*), FaeLga (from *L. gasseri*), FaeLhe (from *L. helveticus*), FaeLjo1 (from *L. johnsonii*), FaeLjo2 (from *L. johnsonii*), and FaeLre (from *L. reuteri*), respectively. The clear halos appeared in the LB medium supplemented with substrate ethyl ferulate and inducer IPTG, when these transformants were inoculated and incubated (Supplementary Figure S1A). While no clear area was found in the plate cultivating the *E. coli* containing the pET-22b vector (Supplementary Figure S1B). These results indicated that these feruloyl esterases were correctly and functionally expressed in the recombinant *E. coli* strains. Furthermore, the feruloyl esterases might also be secreted into extracellular environment like our reported FaeLcr previously (Xu et al., 2019).

### The Secretory Characteristic of *Lactobacillus* Feruloyl Esterases in *E. coli*

**Figure 1:**
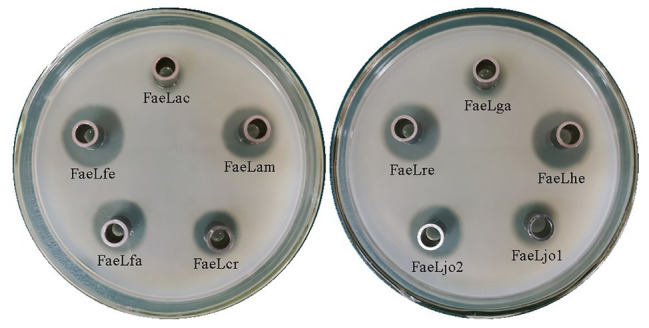
The halos formed by the extracellular cell-free supernatant of the recombinant *Escherichia coli* BL21 (DE3) expressing different *Lactobacillus* feruloyl esterases. FaeLac was derived from *Lactobacillus acidophilus*. FaeLam was derived from *Lactobacillus amylovorus*. FaeLcr was derived from *Lactobacillus crispatus*. FaeLfa was derived from *Lactobacillus farciminis*. FaeLfe was derived from *Lactobacillus fermentum*. FaeLga was derived from *Lactobacillus gasseri*. FaeLhe was derived from *Lactobacillus helveticus*. FaeLjo1 and FaeLjo2 were derived from *Lactobacillus johnsonii*. FaeLre was derived from *Lactobacillus reuteri*.

The cell-free culture supernatants were collected and added into the Oxford cup to test the extracellular feruloyl esterase activity. As shown in Figure 1, the hydrolysis rings were formed by each of the detected samples. While the *E. coli* including the pET-22b vector without any insert showed no clear area (Supplementary Figure S1C). These results suggested that all these *Lactobacillus* feruloyl esterases could be secreted into the extracellular environment of *E. coli*. Furthermore, the difference in the size of the halos indicated the different extracellular feruloyl esterase activities of these *E. coli* strains.

**Figure 2:**
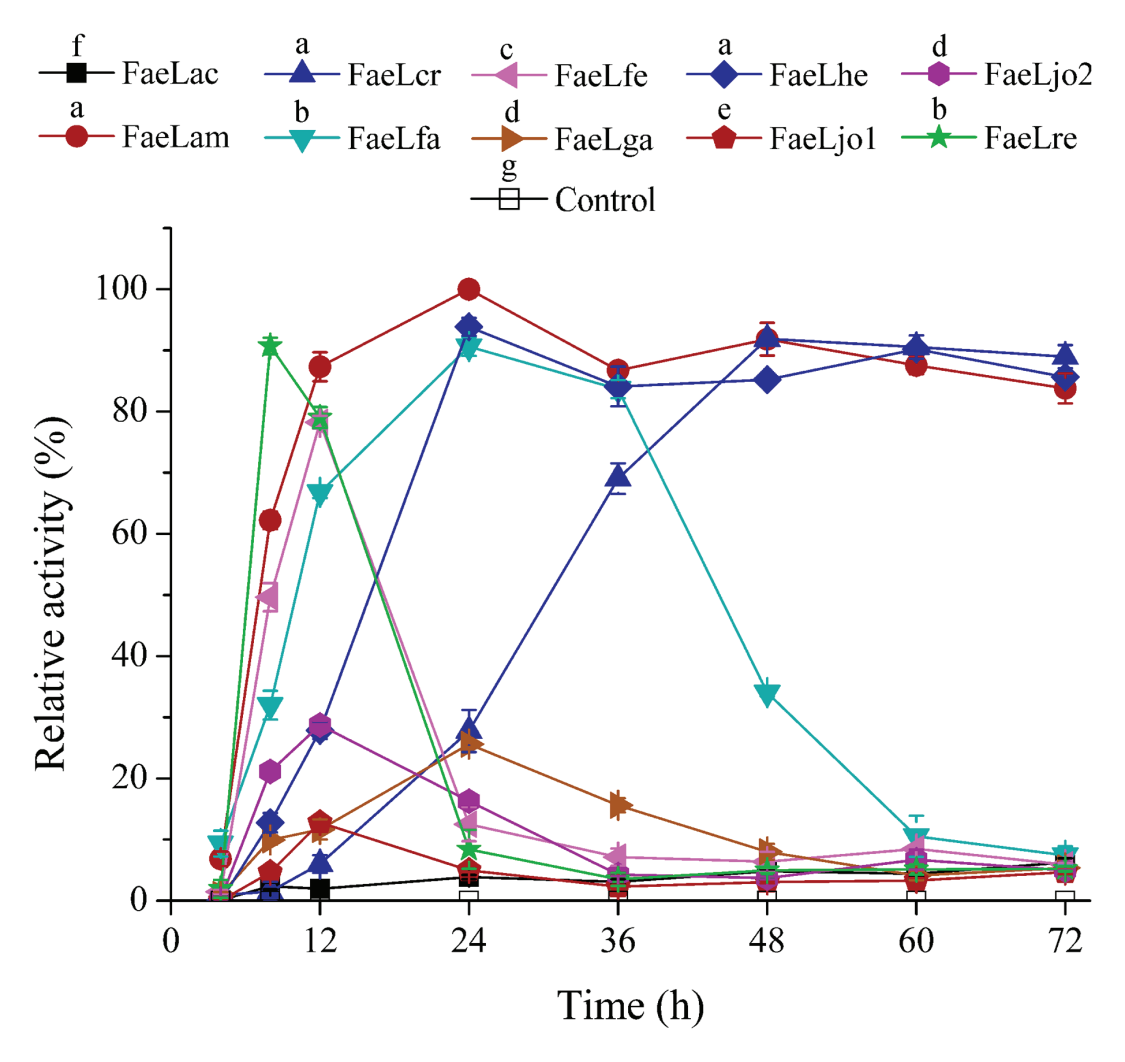
A time course study of extracellular feruloyl esterase activities of these recombinant E. coli BL21 (DE3) strains. Activity was determined at 37°C using para-nitrophenyl ferulate (ρNPF) as substrate. The detected maximal activities of all feruloyl esterases were defined as 100%. Different letters above the icon indicate that significant differences between the maximal activities of each feruloyl esterase at p < 0.05.

The extracellular feruloyl esterase activity changes of these recombinant *E. coli* strains were determined by using ρNPF as substrate. These *E. coli* strains were induced at different temperatures. As shown in Supplementary Table S2, the maximal extracellular activities were detected at 37°C for these feruloyl esterases besides FaeLga. Therefore, the activities of the cell-free supernatant were further measured at time intervals after induction at 37°C. No activity was detected in the supernatant of *E. coli* containing pET-22b, while the extracellular activities of the *E. coli* strains expressing feruloyl esterase exhibited different trends (Figure 2). The extracellular activities of *E. coli* expressing FaeLam and FaeLhe quickly increased and then slightly decreased at the end of the fermentation. The extracellular activities of *E. coli* expressing FaeLfa, FaeLfe, and FaeLre rapidly increased and then dropped to a much lower level. While the extracellular activities of *E. coli* expressing FaeLac, FaeLga, FaeLjo1, and FaeLjo2 increased and decreased less dramatically. These strains reached the maximum extracellular enzyme activity at different time points. The maximal activity of extracellular FaeLre was detected at 8 h. The maximal activities of extracellular FaeLfe, FaeLjo1, and FaeLjo2 were observed at 12 h. The maximal activities of extracellular FaeLac, FaeLam, FaeLfa, FaeLga, and FaeLhe were detected at 24 h. Supplementary Table S3 shows the maximal extracellular activities of these feruloyl esterases.

### Extracellular Protein of Recombinant *E. coli* Strains

**Figure 3:**
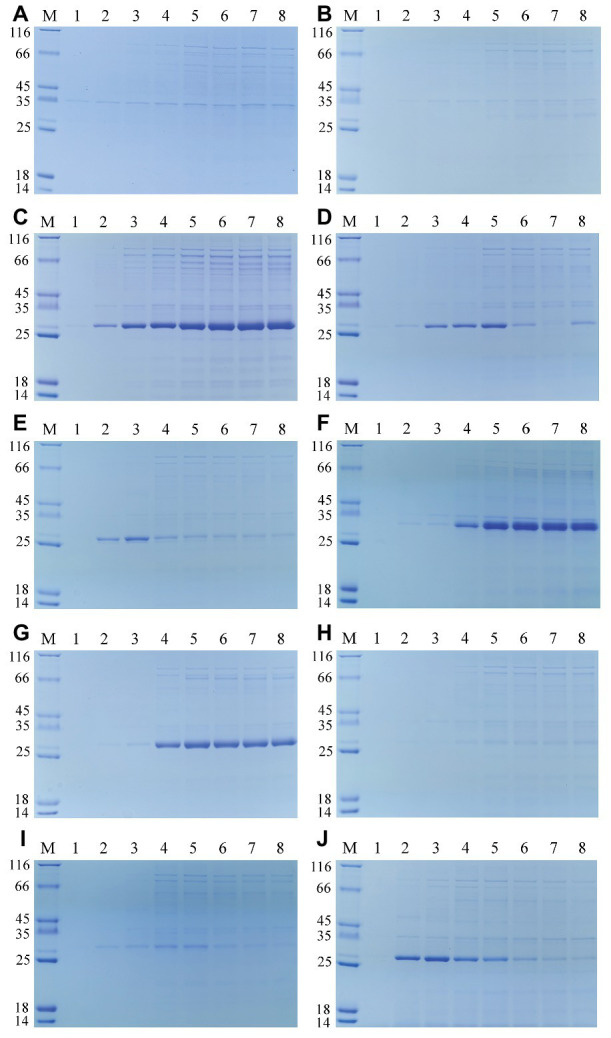
The extracellular protein profiles of the *E. coli* containing pET-22b **(A)**, and expressing feruloyl esterases derived from different *Lactobacillus* species, including *L. acidophilus* **(B)**, *L. amylovorus* **(C)**, *L. farciminis* **(D)**, *L. fermentum* **(E)**, *L. gasseri* **(F)**, *L. helveticus* **(G)**, *L. johnsonii* **(H,I)**, and *L. reuteri* **(J)**. Lane 1–8 represented the samples of 4, 8, 12, 24, 36, 48, 60, and 72 h, respectively.

The extracellular components of these recombinant *E. coli* BL21 (DE3) cultured in LB medium were also collected at time intervals after induction, and then analyzed by using SDS-PAGE. As shown in Figure 3, the secretory protein bands were observed intuitively. Comparing with the extracellular protein profiles of the *E. coli* containing pET-22b (Figure 3A), all the feruloyl esterases could be detected out of the *E. coli* cells but with different content. The extracellular FaeLam and FaeLhe gradually accumulated in the culture medium along with the fermentation (Figures 3C,G). The extracellular FaeLfa, FaeLfe, and FaeLre first increased and then decreased (Figures 3D,E,J). While the extracellular FaeLac, FaeLjo1, and FaeLjo2 exhibited less dramatic changes. Especially, the extracellular FaeLac and FaeLjo1 only showed faint bands at the later stage of fermentation (Figures 3B,H,I). These results were in accordance with the extracellular feruloyl esterase activity changes, indicating that the activities were affected by the secretion and degradation level of feruloyl esterases. However, the FaeLga progressively increased without obvious reduction in extracellular environment (Figure 3F), while the activity displayed on a downward trend after 24 h fermentation. This might be due to the instability of the FaeLga.

Due to the low secretion level of FaeLac, FaeLjo1, and FaeLjo2 as shown in the SDS-PAGE, the presence of the three proteins was further confirmed in cell free supernatants by western blot based on the His_6_ tag. As shown in the Supplementary Figure S2, the positive protein bands of FaeLac, FaeLjo1, and FaeLjo2 appeared in the PVDF membrane. While, no band was observed at the control sample. The extracellular protein concentrations of these recombinant *E. coli* were further measured when the enzymatic activity reached maximum, respectively (Supplementary Table S3). It should be noted that the secretory feruloyl esterases only account for a portion (small or large) of the extracellular proteins.

### Comparison of Cytoplasmic and Extracellular Activities

**Figure 4:**
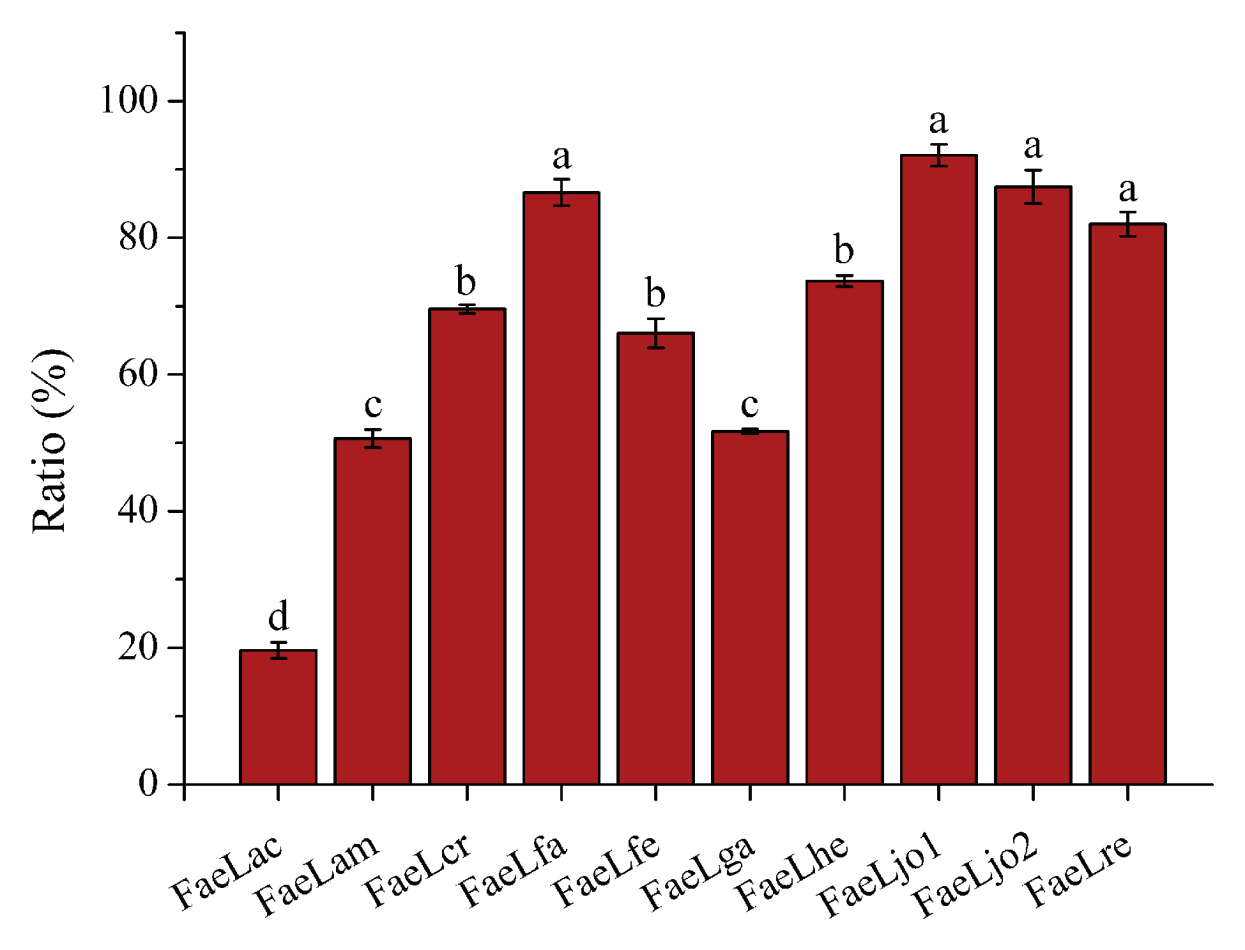
The ratio of extracellular feruloyl esterase activity to total (extracellular plus cytoplasmic) feruloyl esterase activity. Different letters above the column indicate significant differences at p < 0.05.

The extracellularly secretory proteins of these *E. coli* strains were first expressed in cytoplasm and then transported through the protein transport system. Therefore, the feruloyl esterase activities in the cytoplasmic components were also determined using ρNPF as substrate. Figure 4 showed the results of the ratio of extracellular enzyme activity to total (extracellular plus cytoplasmic) enzyme activity when the extracellular activity reached maximum. Except for *E. coli* expressing FaeLac, the extracellular activities account for more than 50% of the total activities. In order to further understand the profile of feruloyl esterases, whole cell proteins and cytoplasmic proteins of recombinant *E. coli* strains were also analyzed by SDS-PAGE. The sampling time was set at the maximum extracellular activity for each feruloyl esterase. As shown in Supplementary Figure S3, it could be clearly observed that these recombinant proteins were produced by *E. coli* in quantities. Furthermore, the secretory feruloyl esterases showed the same molecular weight with the un-transported feruloyl esterases (data not shown), indicating that these proteins were not cleaved or modified during the transport process. However, these *E. coli* showed differences in whole cell and cytoplasmic component. As for FaeLac, FaeLam, FaeLcr, FaeLfa, FaeLga, and FaeLre, the content of intracellularly soluble feruloyl esterases was only slightly lower than that of whole cell. While the majority of FaeLfe, FaeLhe, FaeLjo1, and FaeLjo2 disappeared in the cytoplasmic components.

### Ferulic Acid Production From Agricultural Waste

**Figure 5:**
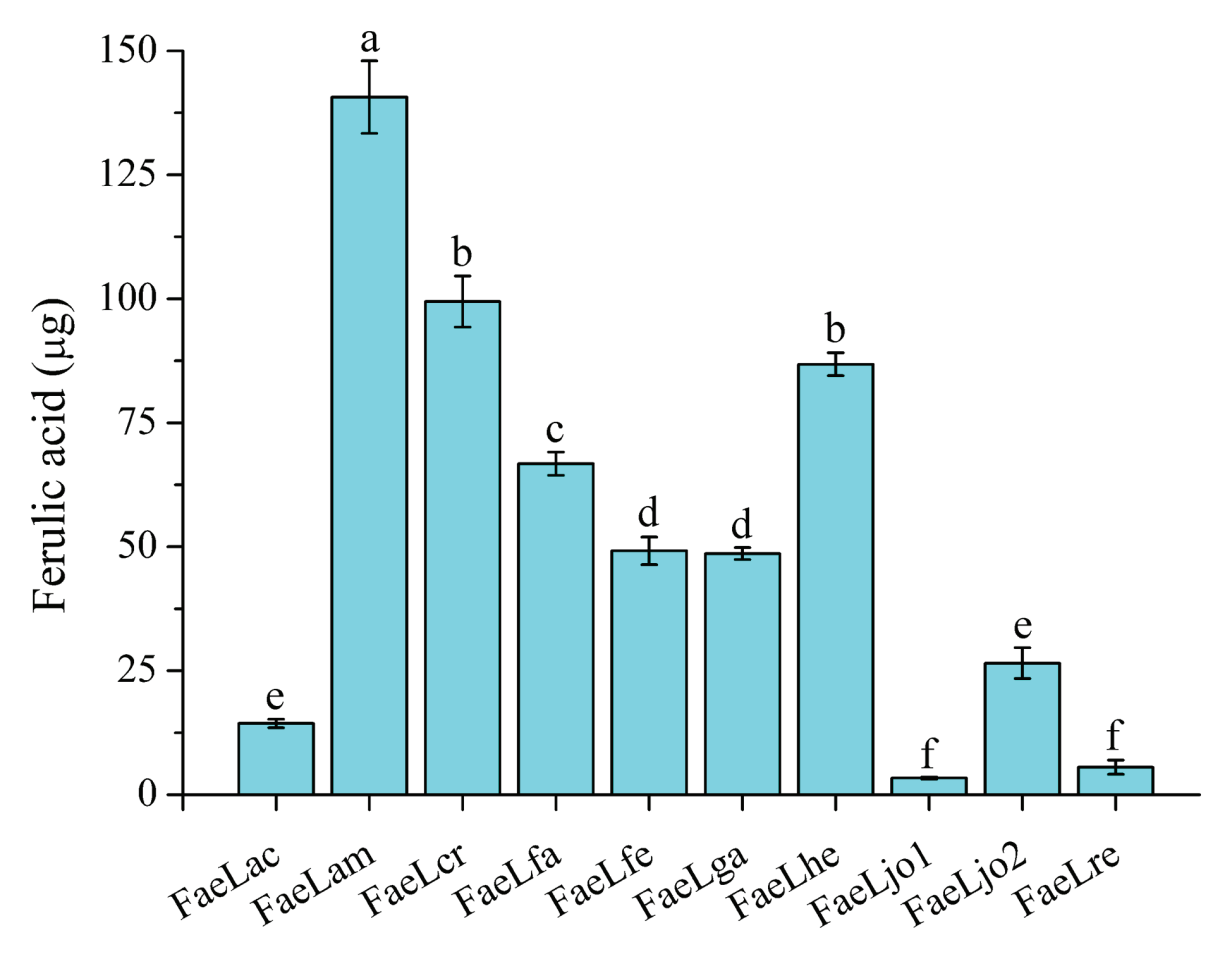
The releasing of ferulic acid from de-starched wheat bran by the recombinant E. coli strains expressing Lactobacillus feruloyl esterases. Different letters above the column indicate significant differences at p < 0.05.

Ferulic acid is widely found in agricultural waste, such as wheat bran and rice bran. Previous experiments were commonly carried out by using purified feruloyl esterase to release ferulic acid from agricultural waste. In the present study, one-step production of ferulic acid from de-starched wheat bran was performed by utilizing the recombinant *E. coli* secreting feruloyl esterase. As shown in Figure 5, all these recombinant strains could hydrolyze the de-starched wheat bran to release ferulic acid. Furthermore, the ferulic acid was also released from de-starched wheat bran by the culture supernatants, suggesting that the feruloyl esterases were also secreted in this medium (Supplementary Figure S4). However, the hydrolytic abilities of those strains were different. The *E. coli* expressing FaeLam displayed the highest hydrolytic activity, while the lowest amount of ferulic acid was obtained by *E. coli* expressing FaeLjo1. In conclusion, these recombinant strains could be directly used for ferulic acid production from agriculture waste, and the hydrolytic ability was in a strain-specific manner. The highest yield of ferulic acid was 140 μg on the basis of 0.1 g de-starched wheat bran after 72 h cultivation of *E. coli* expressing FaeLam.

## Discussion

Lactic acid bacteria are a group of Gram-positive bacteria that can ferment soluble carbohydrates to produce lactic acid. Many species of different genera belong to lactic acid bacteria, in which *Lactobacillus* is an important member (Jarocki et al., 2020). *Lactobacillus* strains are widely distributed in human gut, dairy products, and fermented plant foods (Duar et al., 2017). In recent years, the application value of *Lactobacillus* has been recognized along with the increase of related research. Considering the generally recognized safe status of lactic acid bacteria and the extensive application of feruloyl esterase in the food, cosmetics, and pharmaceutical industries, the feruloyl esterases produced by *Lactobacillus* strains have received increasing attention. Various feruloyl esterase-producing *Lactobacillus* have been isolated from gut or fermented plant products, including those investigated in the present study (Fritsch et al., 2017; Song and Baik, 2017; Xu et al., 2017, 2019; Lai et al., 2019). Their feruloyl esterases have been heterologously expressed in *E. coli* BL21 (DE3), and then purified and characterized previously. However, a new understanding of these feruloyl esterases from another perspective was obtained. Results showed that these feruloyl esterases could be secreted into the extracellular environment of *E. coli*, but had differences in terms of secretion levels and extracellular accumulation. In addition, these recombinant strains could be used to release ferulic acid directly from agricultural waste. These results provided a solid basis for the production of feruloyl esterase and ferulic acid.

Although all of these feruloyl esterases could be secreted out of the *E. coli* cells, three types were classified according to the content and variation of extracellular feruloyl esterases. The first type presented a low secretion level. The second type showed a high secretion level. The third type also demonstrated a high secretion level, but easy degradation. By analysis of whole cell and cytoplasmic proteins, it was found that a part of recombinant feruloyl esterases existed as inclusion bodies. The formation of inclusion bodies might affect their secretion levels, like FaeLjo1 and FaeLjo2. In order to increase extracellular enzyme activity, these feruloyl esterases should be induced with less inducer to weaken the protein expression. The second type feruloyl esterases were ideal candidates for mass production. Furthermore, these feruloyl esterases showed diverse biochemical characteristics (Fritsch et al., 2017; Song and Baik, 2017; Xu et al., 2017, 2019). Therefore, a high expression platform was created to produce feruloyl esterases suitable for different applications. Particularly, the fermentation broths containing feruloyl esterase could be directly used as an additive in pulp and paper or feed industry (Dilokpimol et al., 2016). In addition, the third type feruloyl esterases were highly secreted in the early stage, and then rapidly degraded in the later stage. This might be due to the extracellular protease produced by *E. coli* (Islam et al., 2016). Construction of protease-resistant mutants *via* predicting the action site of protease provides a method to prevent the feruloyl esterase degradation.

Ferulic acid accounts for up to 3% of the dry weight of cells in plants (Lau et al., 2020). Since ferulic acid usually binds to macromolecules such as hemicellulose or lignin in the plant cell walls, only the secretory feruloyl esterase of microorganisms can approach these substrates to release ferulic acid. Therefore, the purified enzymes were often used in previous studies. Four *Lactobacillus* feruloyl esterases were heterologously expressed in *E. coli*, and purified to produce ferulic acid from corn stover (Xu et al., 2017). The feruloyl esterase PcFAE1 of *Penicillium chrysogenum* 31B was overexpressed in *Pichia pastoris* KM71H and then purified to release ferulic acid from natural substrates (Phuengmaung et al., 2019). In the light of the secretory expression of these feruloyl esterases, the direct use of the recombinant *E. coli* strain facilitated the ferulic acid production, because of the saving of enzyme purification process. *Escherichia coli* expressing FaeLam showed the excellent performance with the maximum releasing amount of 140 μg ferulic acid from 0.1 g de-starched wheat bran. It should be noted that the hydrolysis experiments were performed at a constant temperature of 37°C in order to accommodate the growth of *E. coli*. However, most of the feruloyl esterases reached maximum secretion activity within 24 h. In the future, variable temperature fermentation by changing to the optimum temperature of enzymatic activity after 24 h can be tried to increase the yield or shorten the fermentation time.

One of the questions raised by this study is whether these *Lactobacillus* themselves are capable of secreting feruloyl esterase. *Lactobacillus* strains have been used as probiotics in fermented food to improve human health (Han et al., 2020). Those capable of producing feruloyl esterase have greater potential for application. Because the feruloyl esterase can release ferulic acid bound to macromolecules such as hemicellulose and lignin, when the plant-based food is mixed with *Lactobacillus* strains, the produced ferulic acid enhances the probiotic effects (Westfall et al., 2019). The above idea is based on the secretory expression of feruloyl esterase in *Lactobacillus*. However, the localization of feruloyl esterase in *Lactobacillus* is controversial. Lai et al. (2019) and Esteban-Torres et al. (2013) supported *Lactobacillus* feruloyl esterase as intracellular enzyme. Because no signal peptide sequence was predicted in these feruloyl esterases, and the extracellular components of *Lactobacillus plantarum* and *L. johnsonii* cultures were not capable of degrading the model substrate of feruloyl esterase. On the contrary, there were reports that the extracellular feruloyl esterase activity was detected and the ferulic acid was released when natural substrates such as barley were fermented by *Lactobacillus* strains (Hole et al., 2012). In the present study, we showed that all these *Lactobacillus* feruloyl esterases without predictable signal peptide sequences could be secreted into the extracellular environment of *E. coli*. This motivated us to explore the secretion of feruloyl esterase in their natural host in the future.

The other question is how these feruloyl esterases are transported by *E. coli*. *Escherichia coli* have been used as a cell factory to produce a variety of enzymes and medical proteins due to its clear genetic background and sophisticated protein expression control tools. However, the protein expressed by *E. coli* is usually located intracellularly. Obtaining a desired product often requires a complicated purification process. The Sec and Tat are the typical protein secretion pathways in *E. coli*. These two pathways are dependent on typical signal peptide sequences at the N-terminus of the proteins (Natale et al., 2008). However, no predictable signal peptide sequences were found in these *Lactobacillus* feruloyl esterases, indicating that they were transported by a novel protein secretion mechanism of *E. coli*. There were several studies concerning the atypical secretion of proteins. Novel secretory mechanisms have been excavated in *E. coli*, including Type III and Type VI secretion systems (Slater et al., 2018; Navarro-Garcia et al., 2019). Furthermore, the secretion mechanisms of several heterogeneous proteins in *E. coli* were explored by researchers. Su et al. (2013) reported that *Thermobifida fusca* cutinase could hydrolyze the cell membrane of *E. coli*, leading to the cell leakage. Gao et al. (2015) found that the N-terminus of *Bacillus* sp. cellulase could act as the atypical signal peptide for transportation by *E. coli*. Yang et al. (2018) proved that the conformational signal of *Paecilomyces thermophila* β-1, 4-xylosidase played important role for secretion in *E. coli*. These results suggested that the secretion mechanism was specific for different proteins. Nevertheless, the understanding of the atypical protein secretion pathway provides a new solution for the secretory expression of foreign proteins. Recombinant proteins had been successfully secreted by these pathways in *E. coli* (Green et al., 2019). Therefore, the secretion mechanism of feruloyl esterase can broaden the means of protein secretion in *E. coli*. The further work can be carried out by investigation of the structure or sequence basis of the feruloyl esterase for recognition, and exploration of the related protein required for the feruloyl esterase transportation in *E. coli*.

In conclusion, the 10 *Lactobacillus* feruloyl esterases could be secreted into extracellular environments when expressed in *E. coli* BL21 (DE3). However, they also showed differences in terms of secretion levels and extracellular accumulation. Recombinant *E. coli* strains expressing feruloyl esterase of *L. amylovorus*, *L. crispatus*, and *L. helveticus* displayed high secretion levels and stable extracellular activity. Furthermore, these recombinant strains could be used to release ferulic acid directly from agricultural waste. The maximal production was obtained by the *E. coli* expressing *L. amylovorus* feruloyl esterase. These results provided a solid basis for the production of feruloyl esterase and ferulic acid.

## Data Availability Statement

The original contributions presented in the study are included in the article/Supplementary Material, further inquiries can be directed to the corresponding authors.

## Author Contributions

ZX and JK conceived and designed the experiments. ZX and SZ carried out the experimental work. ZX, JK, TW, and XL wrote and revised the manuscript. All authors read and approved the final manuscript.

### Conflict of Interest

The authors declare that the research was conducted in the absence of any commercial or financial relationships that could be construed as a potential conflict of interest.

## Footnotes

^1^http://www.cbs.dtu.dk/services/SignalP/

^2^http://www.cbs.dtu.dk/services/TatP/
