# Supplementary material for: Comparison of Enzyme Secretion and Ferulic Acid Production by Escherichia coli Expressing Different Lactobacillus Feruloyl Esterases
Source: Front Microbiol. 2020 Nov 3;11:568716. doi: 10.3389/fmicb.2020.568716 (PMC7732493; doi:10.3389/fmicb.2020.568716)
Supplement: Supplementary file 1 [file Data_Sheet_1.docx]

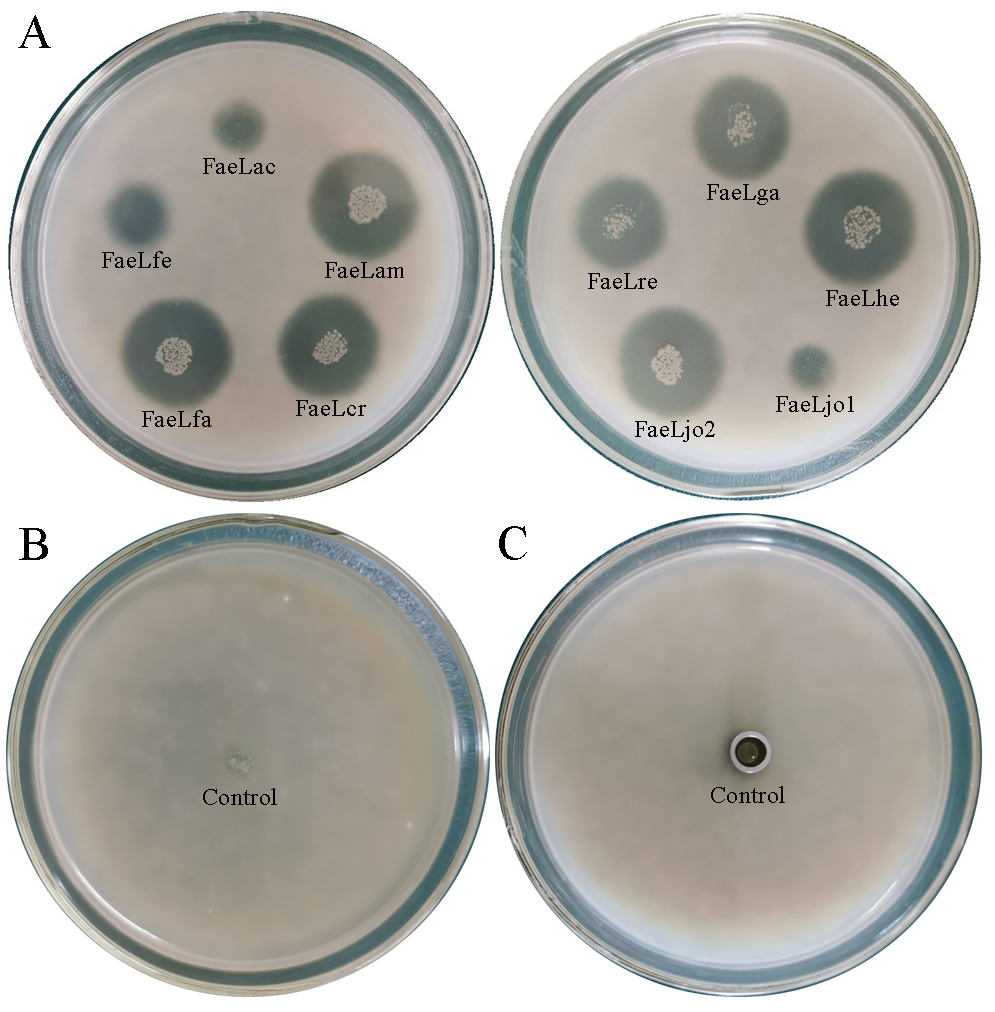


**Figure S1.** The halos formed by the recombinant *E. coli* BL21(DE3) expressing different *Lactobacillus* feruloyl esterases (A), and *E. coli* BL21(DE3) containing pET-22b as negative control (B), and the extracellular cell-free supernatant of the negative control (C). FaeLac was derived from *Lb. acidophilus*. FaeLam was derived from *Lb. amylovorus*. FaeLcr was derived from *Lb. crispatus*. FaeLfa was derived from *Lb. farciminis*. FaeLfe was derived from *Lb. fermentum*. FaeLga was derived from *Lb. gasseri*. FaeLhe was derived from *Lb. helveticus*. FaeLjo1 and FaeLjo2 were derived from *Lb. johnsonii*. FaeLre was derived from *Lb. reuteri*.


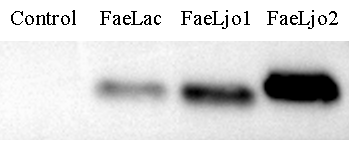


**Figure S2.** The confirmation of the secreted FaeLac, FaeLjo1 and FaeLjo2 by western blot analysis. The extracellular component of *E. coli* containing pET-22b was used as negative control.


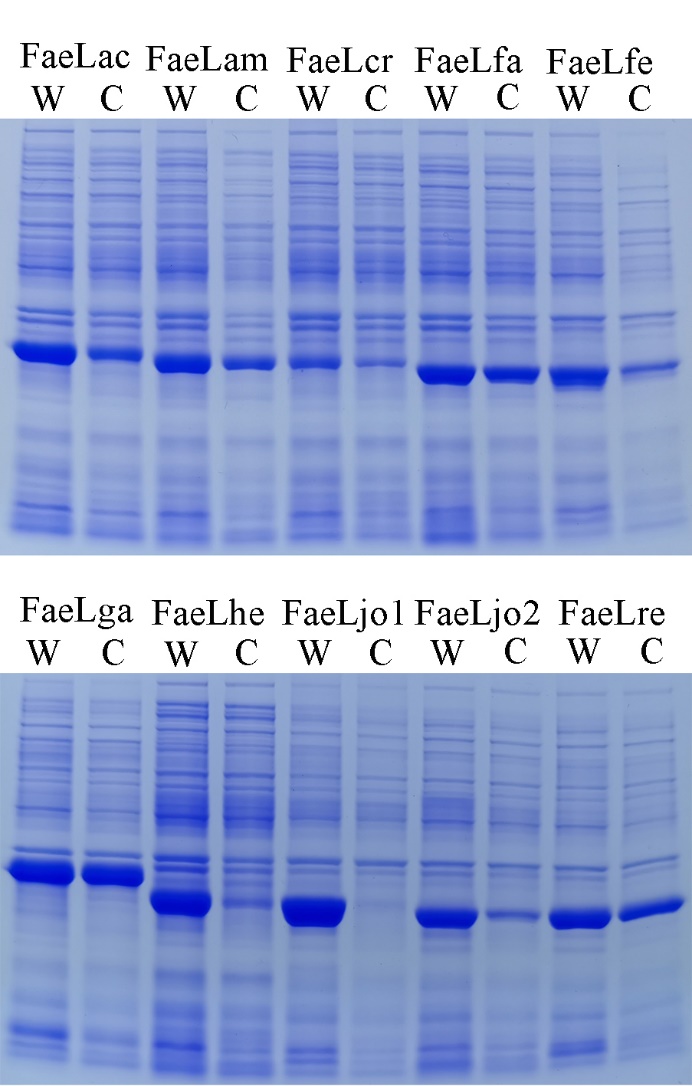


**Figure S3.** The whole cell (W) and cytoplasmic component (C) of recombinant *E. coli* expressing feruloyl esterases derived from different *Lactobacillus* species.





**Figure S4**. The releasing of ferulic acid from de-starched wheat bran by the culture supernatants of recombinant *E. coli* strains expressing *Lactobacillus* feruloyl esterases. Different letters above the column indicate significant differences at p < 0.05.

**Table S1.** Oligonudeotide primers used in this study

| Primer | Sequence(5’-3’) | Template |
| --- | --- | --- |
| FaeLac-F | taagaaggagatatacatATGTCTCGCATTACAATTG | *Lb. acidophilus* |
| FaeLac-R | gtggtggtggtggtgctcgagAAATAGGGGCTTCAAAAATTC |  |
| FaeLam-F | taagaaggagatatacatATGTCCCGCATTACAATTG | *Lb. amylovorus* |
| FaeLam-R | gtggtggtggtggtgctcgagGAATAATGGTTTTAAAAATTG |  |
| FaeLfa-F | taagaaggagatatacatATGAAAGTAGAAATTAAAC | *Lb. farciminis* |
| FaeLfa-R | gtggtggtggtggtgctcgagATCCAACATGAATTTTGTC |  |
| FaeLfe-F | taagaaggagatatacatATGGAAGTTGCAATCAAG | *Lb. fermentum* |
| FaeLfe-R | gtggtggtggtggtgctcgagCGGTTTAAGAAATCGGCCAC |  |
| FaeLga-F | taagaaggagatatacatATGGCAACAATTACAATTG | *Lb. gasseri* |
| FaeLga-R | gtggtggtggtggtgctcgagAAAAGTATTATTATCTTGTAAAAAT |  |
| FaeLhe-F | taagaaggagatatacatATGTCCCGCATTACGATTGAAAGA | *Lb. helveticus* |
| FaeLhe-R | gtggtggtggtggtgctcgagAAACGCAGGTTTTAAAAATTGCG |  |
| FaeLjo1-F | taagaaggagatatacatATGGAGACTACAATTAAACGTG | *Lb. johnsonii* |
| FaeLjo1-R | gtggtggtggtggtgctcgagTTTTATTAAAAACTCACCAAC |  |
| FaeLjo2-F | taagaaggagatatacatATGGCAACAATTACACTTG | *Lb. johnsonii* |
| FaeLjo2-R | gtggtggtggtggtgctcgagAAACGCATTATTATTCTG |  |
| FaeLre-F | taagaaggagatatacatATGGAAATAACAATCAAACG | *Lb. reuteri* |
| FaeLre-R | gtggtggtggtggtgctcgagATTTTTTAAAAAGTTAGCTAC |  |

**Table S2.** The extracellular feruloyl esterase activity of the recombinant *E. coli* incubated at different temperatures

| Feruloyl esterase | 25 °C | 30 °C | 37 °C |
| --- | --- | --- | --- |
| Control | ND | ND | ND |
| FaeLac | 11.8 ± 0.8^c^ | 41.0 ± 4.4^b^ | 100.0 ± 1.2^a^ |
| FaeLam | 8.5 ± 0.7^c^ | 39.7 ± 1.6^b^ | 100.0 ± 4.0^a^ |
| FaeLcr | 24.4 ± 1.7^c^ | 84.9 ± 2.4^b^ | 100.0 ± 2.3^a^ |
| FaeLfa | 20.8 ± 1.3^c^ | 52.0 ± 3.0^b^ | 100.0 ± 3.9^a^ |
| FaeLfe | 2.9 ± 0.5^b^ | 93.4 ± 2.2^a^ | 100.0 ± 2.0^a^ |
| FaeLga | 51.8 ± 1.7^b^ | 100.0 ± 3.5^a^ | 87.7 ± 2.3^a^ |
| FaeLhe | 3.8 ± 1.0^c^ | 29.7 ± 0.9^b^ | 100.0 ± 3.4^a^ |
| FaeLjo1 | 38.2 ± 1.2^c^ | 53.8 ± 1.8^b^ | 100.0 ± 2.1^a^ |
| FaeLjo2 | 57.3 ± 1.5^b^ | 86.0 ± 2.9^a^ | 100.0 ± 3.3^a^ |
| FaeLre | 8.1 ± 0.7^c^ | 15.5 ± 1.1^b^ | 100.0 ± 2.7^a^ |

These strains were incubated at 25 °C, 30 °C and 37 °C for 12 h, respectively. The *E. coli* containing pET-22b was used as negative control. Extracellular activity was determined in 100 mM sodium phosphate buffer (pH 7.0) at 37 °C using 1 mM ρNPF as substrate. Values are means ± standard deviation, n = 3. ND means no detectable activity. The detected maximal activities for each feruloyl esterase were defined as 100 %. Different letters in the same row indicate significant differences at p < 0.05.

**Table S3.** The extracellular enzymatic activity and protein concentration of the recombinant *E. coli* when the maximal activities were detected

| Feruloyl esterase | Maximal activity detected at (h) | Extracellular enzymatic activity (U/L) | Extracellular protein concentration (mg/L) |
| --- | --- | --- | --- |
| FaeLac | 24 | 88.9 ± 8.1^f^ | 51.5 ± 6.3^c^ |
| FaeLam | 24 | 2306.0 ± 23.7^a^ | 219.0 ± 11.0^a^ |
| FaeLcr | 48 | 2118.5 ± 33.8^a^ | 201.7 ± 4.1^a^ |
| FaeLfa | 24 | 2089.8 ± 35.1^b^ | 93.4 ± 10.9^b^ |
| FaeLfe | 12 | 1804.3 ± 24.3^c^ | 106.1 ± 12.6^b^ |
| FaeLga | 24 | 590.4 ± 11.1^d^ | 146.6 ± 6.9^b^ |
| FaeLhe | 24 | 2164.0 ± 34.1^a^ | 135.7 ± 1.8^b^ |
| FaeLjo1 | 12 | 294.9 ± 24.3^e^ | 50.0 ± 3.7^d^ |
| FaeLjo2 | 12 | 662.5 ± 14.9^d^ | 77.7 ± 4.2^c^ |
| FaeLre | 8 | 2092.3 ± 31.2^b^ | 149.5 ± 8.5^b^ |

The *E. coli* strains were incubated at 37 °C for 72 h. Extracellular activity was determined in 100 mM sodium phosphate buffer (pH 7.0) at 37 °C using 1 mM ρNPF as substrate. Values are means ± standard deviation, n = 3. Different letters in the same column indicate significant differences at p < 0.05.
